# Supplementary material for: BRAFV600E cooperates with CDX2 inactivation to promote serrated colorectal tumorigenesis
Source: eLife. 2017 Jan 10;6:e20331. doi: 10.7554/eLife.20331 (PMC5268782; doi:10.7554/eLife.20331)
Supplement: Figure 1—source data 1. — DOI: http://dx.doi.org/10.7554/eLife.20331.003 [file elife-20331-fig1-data1.docx]

| **Figure 1 – source data 1. Clinicopathologic features, BRAF mutation status, and CDX2 expression in 36 human serrated morphology CRCs** | | | | | | | | | | |
| --- | --- | --- | --- | --- | --- | --- | --- | --- | --- | --- |
|  | | |  | CDX2 (+) | CDX2(-) | *p value |  | BRAF mut | BRAF wt | *p value |
| **Patient age** | | | |  |  |  |  |  |  |  |
|  | | age>68 | | 7 | 12 | 0.736 |  | 11 | 8 | 0.978 |
|  | | age<67 | | 8 | 9 |  |  | 9 | 8 |  |
| **Sex** | | | |  |  |  |  |  |  |  |
|  | | Female | | 10 | 12 | 0.732 |  | 11 | 11 | 0.501 |
|  | | Male | | 5 | 9 |  |  | 9 | 5 |  |
| **Tumor location** | | | |  |  |  |  |  |  |  |
|  | Right | | | 5 | 10 | 0.501 |  | 9 | 6 | 0.741 |
|  | Cecum | | | 1 | 4 |  |  | 4 | 1 |  |
|  | Ascending colon | | | 1 | 4 |  |  | 3 | 2 |  |
|  | Transverse colon | | | 3 | 2 |  |  | 2 | 3 |  |
|  | Left | | | 10 | 11 |  |  | 11 | 10 |  |
|  | Descending colon | | | 0 | 0 |  |  | 0 | 0 |  |
|  | Sigmoid colon | | | 1 | 3 |  |  | 3 | 1 |  |
|  | Rectum | | | 9 | 8 |  |  | 8 | 9 |  |
| **Histology** | | | |  |  |  |  |  |  |  |
|  | | Serrated | | 13 | 17 | 0.550 |  | 16 | 14 | 0.770 |
|  | | Mucinous | | 2 | 2 |  |  | 3 | 1 |  |
|  | | Trabecular | | 0 | 2 |  |  | 1 | 1 |  |
|  | |  |  |  |  |  |  |  |  |  |
|  | | Mucin production (+) | | 9 | 12 | 0.661 |  | 15 | 6 | 0.492 |
|  | | (-) | | 6 | 9 |  |  | 5 | 10 |  |
|  | |  |  |  |  |  |  |  |  |  |
|  | | Dirty necrosis (+) | | 5 | 2 | 0.103 |  | 1 | 6 | 0.030 |
|  | | (-) | | 10 | 19 |  |  | 19 | 10 |  |
| **Genetic status** | | | |  |  |  |  |  |  |  |
|  | | BRAF^V600E^ mutant | | 5 | 15 | 0.041 |  |  |  |  |
|  | | wild type | | 10 | 6 |  |  |  |  |  |
|  | | Total | | 15 | 21 |  |  | 20 | 16 |  |
| *p values are determined by Fisher’s exact test (for 2X2 table) and Mantel-Haenszel Chi-Square test of association (for 3X2 table). | | | | | | | | | | |
