# Supplementary material for: BRAFV600E cooperates with CDX2 inactivation to promote serrated colorectal tumorigenesis
Source: eLife. 2017 Jan 10;6:e20331. doi: 10.7554/eLife.20331 (PMC5268782; doi:10.7554/eLife.20331)
Supplement: Figure 2—source data 2. — DOI: http://dx.doi.org/10.7554/eLife.20331.009 [file elife-20331-fig2-data2.docx]

| **Ear tag** | **T1** | | **T2** | | **T3** | | **T4** | | **T5** | | **T6** | | **Invasion (n/N)** |
| --- | --- | --- | --- | --- | --- | --- | --- | --- | --- | --- | --- | --- | --- |
|  | **Size (mm,LxH)** | **Invasion** | **Size (mm,LxH)** | **Invasion** | **Size (mm,LxH)** | **Invasion** | **Size (mm,LxH)** | **Invasion** | **Size (mm,LxH)** | **Invasion** | **Size (mm,LxH)** | **Invasion** |  |
| **15310** | 4.5x1.5 | No | 5x1.5 | No | 4x1 | No | 8.5x3 | Yes | 4x4 | No | 4.5x1 | No | 1/6 |
| **15911** | 4x2.5 | Yes | 3x2 | No | 4.5x3.5 | Yes | 5x7 | No | 5.5x5 | Yes |  |  | 3/5 |
| **15998** | 4.5x2 | No | 12.5x4.5 | Yes | 4.5x1.5 | No |  |  |  |  |  |  | 1/3 |
| **16011** | 21x2 | No | 8x5 | Yes |  |  |  |  |  |  |  |  | 1/2 |
| **16031** | 6x4.5 | Yes | 8x3 | No | 6x3 | No | 10.5x4.5 | Yes | 11x4.5 | Yes | 13x3 | No | 3/6 |
| **16034** | 5x2 | No | 2x1 | No | 11x4 | No | 13x2.5 | Yes | 9x2.5 | No |  |  | 1/5 |
| **20238** | 6x11 | Yes | 8x7 | No | 10x5 | No | 6x4 | No |  |  |  |  | 1/4 |

**Figure 2-source data 2. Tumor size and invasion in *CDX2-CreER^T2^ Cdx2^fl/fl^ Braf^CA^*** **mice post TAM Injection.**
