# Supplementary material for: BRAFV600E cooperates with CDX2 inactivation to promote serrated colorectal tumorigenesis
Source: eLife. 2017 Jan 10;6:e20331. doi: 10.7554/eLife.20331 (PMC5268782; doi:10.7554/eLife.20331)
Supplement: Figure 5—source data 2. — DOI: http://dx.doi.org/10.7554/eLife.20331.018 [file elife-20331-fig5-data2.docx]

**Figure 5-source data 2. Comparison of mouse genes up- and down-regulated in *Cdx2^-/-^Braf^V600E^* tumors to gene signatures from human CRCs with low Cdx2 expression and *Braf^V600E^* mutation (TCGA data) and human serrated carcinomas.**

| **TCGA data** |  | 18 V600E+CDX2lo vs. 104 WT+CDX2hi tumors, *P*<.01, FC>1.3 | | | | | | | |
| --- | --- | --- | --- | --- | --- | --- | --- | --- | --- |
|  |  | up | neither | down | total |  | Observed/Expected | | |
| Cdx2 by Braf | up | 390 | 772 | 112 | 1274 |  | 1.75 |  | 0.59 |
| interaction | neither | 2274 | 9396 | 2054 | 13724 |  |  |  |  |
| *P*<.01, FC>1.3 | down | 76 | 378 | 178 | 632 |  | 0.69 |  | 1.88 |
|  | total | 2740 | 10546 | 2344 | 15630 |  |  |  |  |
| Mantel-Haenszel Chi-Square test of association, *P*-value: | | | | | 4.5E-49 |  |  |  |  |

| **Finnish data** |  | GSE4045, 8 serrated vs. 29 conventional tumors, *P*<.01, FC>1.3 | | | | | | | |
| --- | --- | --- | --- | --- | --- | --- | --- | --- | --- |
|  |  | up | neither | down | total |  | Observed/Expected | | |
| Cdx2 by Braf | up | 66 | 879 | 25 | 970 |  | 2.26 |  | 0.40 |
| interaction | neither | 241 | 8417 | 623 | 9281 |  |  |  |  |
| *P*<.01, FC>1.3 | down | 16 | 424 | 36 | 476 |  | 1.12 |  | 1.19 |
|  | total | 323 | 9720 | 684 | 10727 |  |  |  |  |
| Mantel-Haenszel Chi-Square test of association, *P*-value: | | | | | 1.4E-11 |  |  |  |  |

**TCGA data**: We downloaded Illumina Genome Analyzer mRNA-seq normalized reads for 263 primary colorectal adenocarcinoma tumors from Broad GDAC interfaces to TCGA project data (<https://gdac.broadinstitute.org/>) as well as mutation data, which was available for 212 of the samples. We divided the samples into 106 with above median CDX2 expression, 2 of which had BRAF V600E mutations (2%), and 106 with below median expression 18 of which had BRAF V600E mutations (17%, *P*=0.0002, two-sided Fisher Exact Test). After adding 1 to the normalized counts for each gene and log-transforming, we compared the 18 samples that had low CDX2 and V600E mutations to the 104 samples with high CDX2 that were not BRAF mutant, by two-sample T-tests, and selected genes as differing between the two groups if *P* for the test was less than 0.01, and if the fold-difference (“FC”) in means was 1.3 or more (either up or down). This selected 6045 genes out of 20531 total genes. We computed the intersection of this selection to the similar selection we had performed that ask for significant Cdx2 by Braf interactions in our mouse data, using only 1-to-1 best mouse to human homologs from NCBI Homologene build 68. The results are shown in the first table. We observed an excess of genes found “up” in both data-sets as well as “down” in both data-sets as well as too few disagreements (*P*=4.5x10^-49^, Mantel-Haenszel Chi-Square test of association). The identities of all the genes in the intersections, their *P*-values, and fold-changes, can be obtained from our supplementary file in NCBI’s GEO series GSE84650.

**Finnish data**: We obtained expression data for 8 serrated and 29 conventional colorectal cancers from GEO series GSE4045 ([Laiho et al. 2007](#_ENREF_1)), which were assayed with Affymetrix HG_U133A arrays, and log-transformed the data after adding 1 to each probe-set’s estimated expression. We compared the two groups using two-sample T-tests and again selected probe-sets that gave P<0.01 and fold-changes of at least 1.3, yielding 1442 selected probe-sets out of 22283 total (representing 12468 distinct human genes). We again joined to our mouse data using 1-to-1 best homologs and computed the intersections as shown in the second table, which were again significantly better than expected by chance (*P*=1.4x10^-11^). Details are available from our GEO series.
