# Supplementary material for: BRAFV600E cooperates with CDX2 inactivation to promote serrated colorectal tumorigenesis
Source: eLife. 2017 Jan 10;6:e20331. doi: 10.7554/eLife.20331 (PMC5268782; doi:10.7554/eLife.20331)
Supplement: Figure 6—source data 1. — DOI: http://dx.doi.org/10.7554/eLife.20331.023 [file elife-20331-fig6-data1.docx]

| **Figure 6-source data 1. Expression of PDX1, CDX2 and ANXA10 in 395 Human CRCs** | | | | | | | | |
| --- | --- | --- | --- | --- | --- | --- | --- | --- |
|  |  |  | **PDX1** | |  | **ANXA10** | |  |
|  |  |  | **positive** | **negative** | ***p value** | **positive** | **negative** | ***p value** |
| **CDX2** | **positive** |  | 87 | 200 | 0.0013 | 9 | 279 | 5.0X10^-7^ |
|  | **low** |  | 24 | 49 |  | 9 | 64 |  |
|  | **absent** |  | 21 | 14 |  | 8 | 26 |  |
|  | total |  | 132 | 263 |  | 26 | 369 |  |
| *p values determined by Mantel-Haenszel Chi-square test of association | | | | | | | | |
|  |  |  |  |  |  |  |  |  |
