# Supplementary material for: BRAFV600E cooperates with CDX2 inactivation to promote serrated colorectal tumorigenesis
Source: eLife. 2017 Jan 10;6:e20331. doi: 10.7554/eLife.20331 (PMC5268782; doi:10.7554/eLife.20331)
Supplement: Supplementary file 1. — DOI: http://dx.doi.org/10.7554/eLife.20331.029 [file elife-20331-supp1.docx]

| **Supplementary file 1. Antibodies for immunohistochemistry study**  **For Mouse tissue** | | | | | | | | | | |  |  |  |  |  |
| --- | --- | --- | --- | --- | --- | --- | --- | --- | --- | --- | --- | --- | --- | --- | --- |
| **Antibody** | **Animal Species** | **Dilution** | | **Source** | |  |  |  | |  |  |  |  |  |  |
| CDX2 | mouse | | 50x | | GeneTex Cat# GTX90831, RRID:AB_845625 | | | | | |  |  |  |  |  |
| β-catenin | mouse | | 800x | | BD Biosciences Cat# 610153, RRID:AB_397554 | | | | | | | |  |  |  |
| BrdU | mouse | | 500x | | BD Biosciences Cat# 560808, RRID:AB_2033928 | | | | | | | |  |  |  |
| p53 | rabbit | | 2000x | | Vector Laboratories Cat# VP-P956, RRID:AB_2335917 | | | | | | | |  |  |  |
| MUC5AC | mouse | | 100x | | Thermo Fisher Scientific Cat# MA1-21907, RRID:AB_560214 | | | | | | |  |  |  |  |
| PDX1 | mouse | | 100x | | DSHB Cat# F109-D12, RRID:AB_1157903 | | | | | | | |  |  |  |
| ANXA10 | rabbit | | 300x | | Novus Cat# NBP1-90156, RRID:AB_11004664 | | | |  | | | | |  |  |
| Phospho-EGFR  (Tyr845) | rabbit | | 100x | | Thermo Fisher Scientific Cat# 44-784G, RRID:AB_2533752 | | | | | | | |  |  |  |
| Phospho-ERK | rabbit | | 100x | | Cell Signaling Technology Cat# 9101, RRID:AB_331646 | | | | | |  |  |  |  |  |
|  |  |  | |  | |  |  |  | |  |  |  |  |  |  |
| **For Human tissue** | |  | |  | |  |  |  | |  |  |  |  |  |  |
| **Antibody** | **Animal Species** | **Dilution** | | **Source** | |  |  |  | |  |  |  |  |  |  |
| CDX2 | mouse | 50x | | GeneTex Cat# GTX90831, RRID:AB_845625 | | | | | |  |  |  |  |  |  |
| β-catenin | mouse | 800x | | BD Biosciences Cat# 610153, RRID:AB_397554 | | | | | |  |  |  |  |  |  |
| MLH1 | mouse | Diluted | | Agilent Technologies Cat# M364001, RRID:AB_2631352 | | | | | |  |  |  |  |  |  |
| MSH2 | mouse | 20x | | Agilent Technologies Cat# M363901-2, RRID:AB_2631353 | | | | | |  |  |  |  |  |  |
| p16 | mouse | 50x | | BD Biosciences Cat# 550834, RRID:AB_2078446 | | | | | |  |  |  |  |  |  |
| p53 | mouse | 50x | | Leica Microsystems Cat# NCL-L-p53-DO7, RRID:AB_563936 | | | | | |  |  |  |  |  |  |
| MUC5AC | mouse | 50x | | Leica Microsystems Cat# NCL-MUC-5AC, RRID:AB_442113 | | | | | |  |  |  |  |  |  |
| MUC6 | mouse | 50x | | Leica Microsystems Cat# NCL-MUC-6, RRID:AB_442114 | | | | | |  |  |  |  |  |  |
| ANXA10 | rabbit | 300x | | Novus Cat# NBP1-90156, RRID:AB_11004664 | | | | | |  |  |  |  |  |  |
| PDX1 | rabbit | 1000x | | Abcam Cat# ab134150, RRID: AB_2631338 | | | | | |  |  |  |  |  |  |
